# First-principles study on elastic properties of Cu, (Cu_1−_*_x_*,Ni*_x_*)_3_Sn and interfacial mechanical properties of (Cu_1−_*_x_*,Ni*_x_*)_3_Sn/Cu in the lead-free solder joint

**DOI:** 10.3762/bjnano.17.29

**Published:** 2026-03-19

**Authors:** Guomin Hua

**Affiliations:** 1 School of Material Science and Engineering, Jiangsu University, Zhenjiang 212013, Jiangsu, China

**Keywords:** ductility, elastic modulus, interfacial toughness, lead-free solder, work of adhesion

## Abstract

In this study, the elastic properties of Cu and (Cu*_x_*Ni_1−_*_x_*)_3_Sn were calculated to reveal the effects of Ni alloying on the interfacial mechanical properties of (Cu*_x_*Ni_1−_*_x_*)_3_Sn/Cu in lead-free solder joints. The results reveal that, within the thermodynamically stable domain of (Cu*_x_*Ni_1−_*_x_*)_3_Sn, the increase of Ni content can enhance the interfacial mechanical properties of (Cu*_x_*Ni_1−_*_x_*)_3_Sn/Cu, and increase the reliability of the lead-free solder joints. The enhancement mechanism can be attributed to the simultaneous improvements of oriented Young’s modulus and ductility of (Cu*_x_*Ni_1−_*_x_*)_3_Sn, achieved by Ni alloying. But higher Ni content beyond the thermodynamically stable domain of (Cu*_x_*Ni_1−_*_x_*)_3_Sn will deteriorate the interfacial mechanical properties by mechanical or thermodynamic mechanisms and decrease the reliability of the lead-free solder joints. The results presented in this study will not only unveil the effects of Ni alloying on the interfacial properties of lead-free solder joints, but also will provide a guidance for high-performance lead-free solder design by alloying strategies to meet the requirements for electronic device miniaturization and harsh environmental applications.

## Introduction

Due its toxicity, lead has caused serious problems in human health and environmental pollution; thus, the use of lead-containing solder in electronic device packaging has been restricted by legislation [1]. In the last few decades, aiming to replace lead-containing solders, great efforts have been dedicated to develop lead-free solder with respect to cost-effectiveness, wettability, melting point, corrosion resistance, and mechanical and electrical properties [1–2]. A series of binary alloy solders, like Sn-Zn alloys [3], Sn-Cu alloys [4], Sn-Ag alloys [5], and Sn-Bi alloys [6], have been extensively investigated. Moreover, ternary and quaternary alloys have recently received considerable attention, and the Sn-Ag-Cu ternary alloy is considered as a promising candidate to substitute conventional lead-containing solder alloys [7].

Driven by the miniaturization of electronic devices and their widespread application in harsh environments such as high temperature and high humidity, the reliability of solder joints has become a major issue in practice [2,8]. As far as lead-containing solders are concerned, the high quality of solder joints can be attributed to the formation of a continuous Pb layer, serving as a barrier layer to separate the intermetallics in solders from the substrate [9]. In contrast to the barrier layer formed with lead-containing solder, the lead-free solder forms a compact interface between the intermetallics and the substrate without a barrier layer [9]. The compact interface could result in poor resistance to high temperatures and thermal shocks. Therefore, high-lead solder remains the preferred choice for high-temperature applications at present [1].

In view of the fact that Ni and Pb belong to the same group in the periodic table of elements and have similar chemical and electronic properties [10], researchers have been intrigued to enhance the reliability of lead-free solder joints by Ni alloying. For instance, Zhang et al. observed that the corrosion resistance of Sn-Zn solder can be enhanced by alloying with Ni, Cr, Cu, or Ag; the higher corrosion resistance follows the order Ag < Cu < Cr < Ni [11]. El-Taher et al. demonstrated that the ductility and the strength of Sn–3.0Ag–0.5Cu lead-free solders could be enhanced by Ni alloying [12]. Although the beneficial effects of Ni alloying on the properties of lead-free solder have been demonstrated by these investigations, little attention has been paid to the effects of Ni alloying on the interfacial mechanical properties of lead-free solder joints.

Considering the important role the interface between the intermetallics and the substrate plays in the strength and reliability of a solder joint, Gan et al. investigated the formation of Cu_3_Sn and Cu_6_Sn_5_ on a Cu substrate and determined the orientation relationship of ε-Cu_3_Sn/Cu interfaces as (001)_ε_//(111)_Cu_ and [100]_ε_//[−110]_Cu_ [13], that is, the interface was constructed by attaching the (001) facet of ε-Cu_3_Sn to the (111) facet of the Cu substrate and making the [100] axis of ε-Cu_3_Sn parallel to the [−110] axis of Cu substrate. Based on the orientation relationship of the ε-Cu_3_Sn/Cu interface, an attempt to reveal the effects of Ni alloying on the strength and toughness of (Cu_1−_*_x_*Ni*_x_*)_3_Sn/Cu interface has been made in this study. At first, the elastic properties of (Cu_1−_*_x_*Ni*_x_*)_3_Sn and Cu were calculated, followed by evaluation of the intrinsic ductility in terms of elastic moduli. Subsequently, the orientation-dependent Young’s moduli of Cu and (Cu_1−_*_x_*Ni*_x_*)_3_Sn were calculated. Finally, tensile modulus, ultimate tensile stress, work of adhesion, and interfacial toughness of (Cu_1−_*_x_*Ni*_x_*)_3_Sn/Cu were calculated based the interface model with the orientation relationship of (001)_ε_//(111)_Cu_ and [100]_ε_//[−110]_Cu_; the underlying mechanisms responsible for the influence of Ni alloying on the work of adhesion and interfacial toughness are demonstrated.

## Methods

In this study, first-principles calculations within the framework of density functional theory were implemented by the ABINIT package [14]. The norm-conserving pseudopotentials [15] and Perdew–Burke–Ernzerhof generalized gradient approximation (GGA) of the exchange–correlation functional [16] were adopted for the calculation. Regarding the calculations on the intermetallics (Cu*_x_*Ni_1−_*_x_*)_3_Sn, virtual crystal approximation (VCA) was used to construct the virtual atoms standing for the mixture of Cu and Ni atoms, namely, the pseudopotentials of the virtual atoms were constructed by [17]:




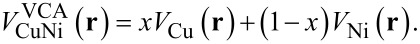




As demonstrated in previous studies [18–21], the VCA could significantly enhance the calculation efficiency without losing the accuracy by reducing the model size of the alloy systems. Considering the phase stability of (Cu*_x_*Ni_1−_*_x_*)_3_Sn [22], the content of Ni was set within the range from 0 to 30 atom %. As far as the calculations of the structure optimizations and elastic properties are concerned, a kinetic energy cutoff of 30 Hartree, a k-point mesh of 8 × 8 × 8 and a potential residual *V*(*r*) of less than 10^−8^ Hartree were used to achieve self-consistent convergence.

Based on the optimized crystal structures, the elastic constants of FCC Cu and orthorhombic (Cu*_x_*Ni_1−_*_x_*)_3_Sn were calculated by finite strain methods, where three and nine deformations were built for Cu and (Cu*_x_*Ni_1−_*_x_*)_3_Sn, respectively, related to the three and nine independent elastic constants corresponding to cubic and orthorhombic crystals, respectively [23]. Strain magnitudes of −0.02, −0.01, 0.0, 0.01, and 0.02 were used to calculated the energy increments of the deformed cells. Via quadratic fits of the relation between the energy increments and the strains, the elastic constants *C*_11_, *C*_12_, and *C*_44_ for Cu and *C*_11_, *C*_22_, *C*_33_, *C*_12_, *C*_13_, *C*_23_, *C*_44_, *C*_55_, and C_66_ for (Cu*_x_*Ni_1−_*_x_*)_3_Sn were extracted. Based on the calculated elastic constants, bulk modulus, shear modulus, Young’s modulus, anisotropy, and Poisson’s ratio of Cu and (Cu*_x_*Ni_1−_*_x_*)_3_Sn were calculated according to Voight–Reuss–Hill bounds [24–25]. Furthermore, from the calculated elastic constants, the orientation-dependent Young’s moduli of Cu and (Cu*_x_*Ni_1−_*_x_*)_3_Sn were calculated.

Based on the orientation relationship of Cu_3_Sn/Cu interfaces [13], namely, (001)_ε_//(111)_Cu_ and [100]_ε_//[−110]_Cu_, the interface was constructed by adhering Cu and (Cu*_x_*Ni_1−_*_x_*)_3_Sn slabs. The Cu slab consisted of four atomic layers, the (Cu*_x_*Ni_1−_*_x_*)_3_Sn slab consisted of three atomic layers, and the thickness of the vacuum layer was 1 nm. Interfacial modulus, ultimate tensile stress, work of adhesion, and the interfacial toughness of (Cu*_x_*Ni_1−_*_x_*)_3_Sn /Cu interfaces were determined by a tensile test along the direction normal to the interface plane, that is, along the *z*-axis. During the tensile deformation, the strain along the *z*-axis was fixed; at the same time, the stresses along the *x*-axis and the *y*-axis were relaxed to less than 0.5 GPa. For the calculations on the interface structure, a kinetic energy cutoff of 30 Hartree, a k-point mesh of 4 × 4 × 1 and a potential residual *V*(*r*) of less than 10^−8^ Hartree were used to achieve self-consistent convergence.

## Results and Discussions

### Elastic properties of Cu and (Cu*_x_*Ni_1−_*_x_*)_3_Sn

**Figure 1:**
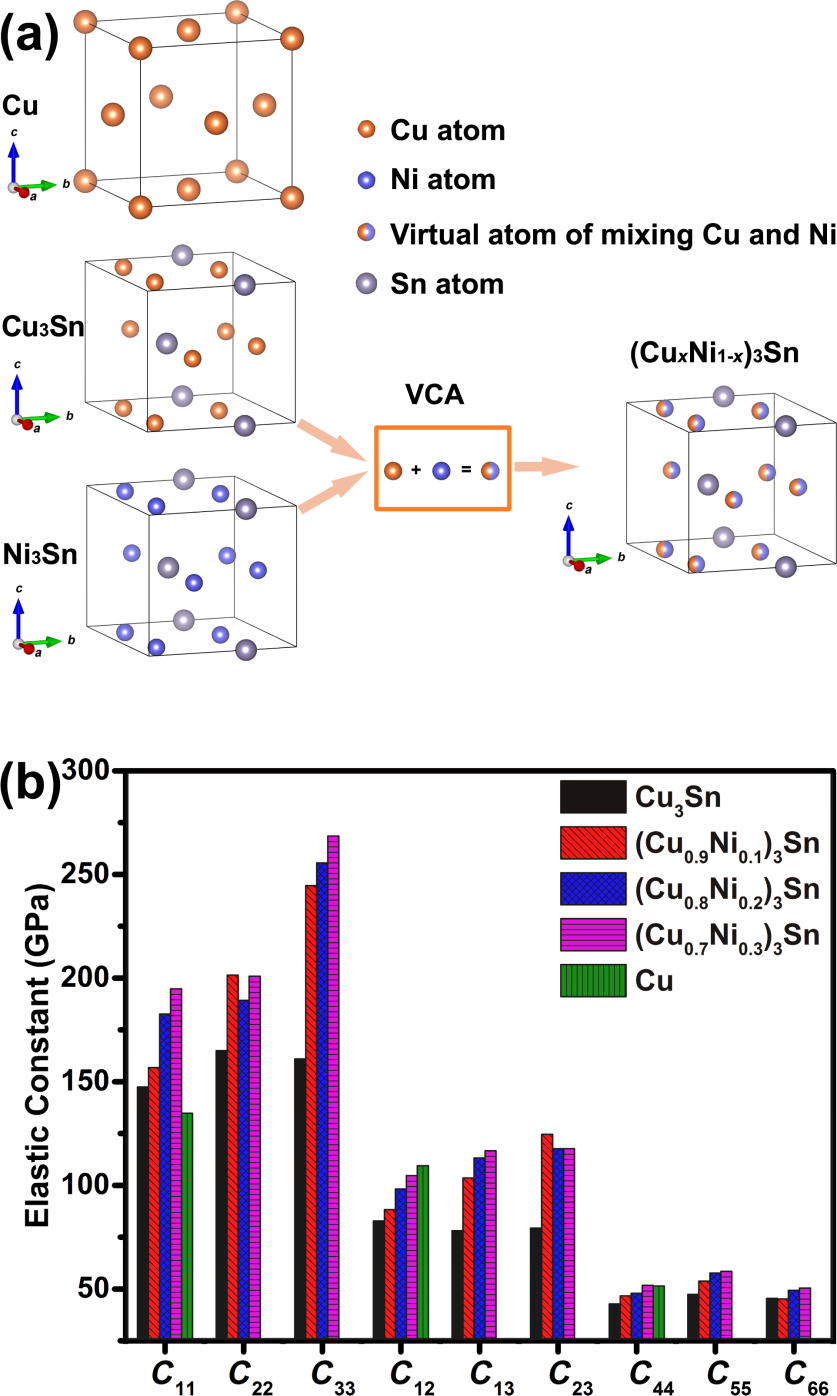
(a) Crystal structures of Cu and (Cu*_x_*Ni_1−_*_x_*)_3_Sn, where the virtual crystal approximation is adopted for (Cu*_x_*Ni_1−_*_x_*)_3_Sn, and the atomic models were plotted using VESTA [28]. This content is not subject to CC BY 4.0. (b) The independent elastic constants, *C*_11_, *C*_22_, *C*_33_, *C*_12_, *C*_13_, *C*_23_, *C*_44_, *C*_55_, and *C*_66_ of (Cu*_x_*Ni_1−_*_x_*)_3_Sn, and the independent elastic constants, *C*_11_, *C*_12_, and *C*_44_ of Cu.

Figure 1a presents the optimized crystal structures of Cu and (Cu*_x_*Ni_1−_*_x_*)_3_Sn, where Cu crystallizes into the face-centered cubic (FCC) structure, and the (Cu*_x_*Ni_1−_*_x_*)_3_Sn crystallizes into an orthorhombic structure. Ni alloying in (Cu*_x_*Ni_1−_*_x_*)_3_Sn is represented by virtual atoms that substitute Cu atoms in Cu_3_Sn, the corresponding pseudopotentials of the virtual atoms were constructed by VCA [17]. As listed in Table 1, the independent elastic constants *C*_11_, *C*_12_ and, C_44_ are 134.8, 109.5, and 51.5 GPa, respectively, for FCC Cu. The calculated elastic constants of Cu are consistent with the measured elastic constants [26]. The independent elastic constants *C*_11_, *C*_22_, *C*_33_, *C*_12_, *C*_13_, *C*_23_, *C*_44_, *C*_55_, and *C*_66_ are 147.5, 165.0, 161.0, 82.9, 78.1, 79.4, 42.8, 47.4, and 45.5 GPa, respectively, for Cu_3_Sn. The calculated elastic constants of Cu_3_Sn are in good agreement with the elastic constants of Cu_3_Sn reported by Pang and coworkers [27]. The dependence of the elastic constants of (Cu*_x_*Ni_1−_*_x_*)_3_Sn on the Ni content are presented in Figure 1b. It can be observed that the tensile elastic constants, *C*_11_, *C*_22_, and *C*_33_, and the orthogonal elastic constants, *C*_12_, *C*_13_, and *C*_23_, were significantly enhanced by the Ni alloying. In contrast, only slight improvements of the shear elastic constants, *C*_44_, *C*_55_, and *C*_66_, were obtained by Ni alloying.

**Table 1:** Space groups, lattice constants, and independent elastic constants of Cu and (Cu*_x_*Ni_1−_*_x_*)_3_Sn; Voigt-type bulk modulus *B*_V_ and Reuss-type bulk modulus *B*_R_; Voigt-type shear modulus *G*_V_ and Reuss-type shear modulus *G*_R_. The data presented in parentheses are given for comparison.

	Cu	Cu_3_Sn	(Cu_0.9_Ni_0.1_)_3_Sn	(Cu_0.8_Ni_0.2_)_3_Sn	(Cu_0.7_Ni_0.3_)_3_Sn

space group	*Fm−*3*m*	*Pmmn*	*Pmmn*	*Pmmn*	*Pmmn*
lattice constants (Å)	*a* = 3.665 (3.615)^a^ *b* = 3.665 (3.615)^a^ *c* = 3.665 (3.615)^a^	*a* = 5.595 (5.618)^b^ *b* = 4.403 (4.367)^b^ *c* = 4.831 (4.835)^b^	*a* = 5.549 *b* = 4.367 *c* = 4.791	*a* = 5.523 *b* = 4.346 *c* = 4.768	*a* = 5.492 *b* = 4.321 *c* = 4.742
*C*_11_ (GPa)	134.8 (169.1)^a^	147.5 (154.6)^b^	156.8	182.7	194.8
*C*_22_ (GPa)	—	165.0 (173.7)^b^	201.5	189.2	200.9
*C*_33_ (GPa)	—	161.0 (148.2)^b^	244.6	255.6	268.5
*C*_12_ (GPa)	109.5 (122.2)^a^	82.9 (78.9)^b^	88.3	98.2	104.7
*C*_13_ (GPa)	—	78.1 (76.5)^b^	103.5	113.2	116.7
*C*_23_ (GPa)	—	79.4 (95.1)^b^	124.6	117.7	117.7
*C*_44_ (GPa)	51.5 (75.4)^a^	42.8 (50.2)^b^	46.7	48.0	51.7
*C*_55_ (GPa)	—	47.4 (44.2)^b^	53.8	57.7	58.5
*C*_66_ (GPa)	—	45.5 (55.0)^b^	45.3	49.4	50.4
*B*_V_ (GPa)	117.9	106.0	137.3	142.9	149.2
*B*_R_ (GPa)	117.9	105.8	128.4	137.8	145.0
*G*_V_ (GPa)	35.9	42.7	48.2	50.9	53.8
*G*_R_ (GPa)	23.1	42.2	47.4	50.1	53.0

^a^The experimental lattice constants and elastic constants of Cu are cited from [26]. ^b^The calculated lattice constants and elastic constants of Cu_3_Sn are cited from [27].

Using the calculated elastic constants of Cu and (Cu*_x_*Ni_1−_*_x_*)_3_Sn, the average bulk modulus, shear modulus, Young’s modulus, universal anisotropy, and Poisson’s ratio were calculated according to the Voigt–Reuss–Hill approximations [25]. For FCC Cu, the Voigt-type bulk modulus *B*_V_ and shear modulus *G*_V_, and the Reuss-type bulk modulus *B*_R_ and shear modulus *G*_R_, can be calculated from *C*_11_, *C*_12_, and *C*_44_ as [25]:


[1] $$B_{\text{V}}=B_{\text{R}}=\left(C_{11}+2C_{12}\right)/3,$$

[2] $$G_{\text{V}}=\left(C_{11}-C_{12}+3C_{44}\right)/5,$$

[3] $$G_{\text{R}}=5\left(C_{11}-C_{12}\right)C_{44}/\left[4C_{44}+3\left(C_{11}-C_{12}\right)\right].$$

For orthorhombic (Cu*_x_*Ni_1−_*_x_*)_3_Sn, the Voigt-type bulk modulus *B*_V_ and shear modulus *G*_V_, and the Reuss-type bulk modulus *B*_R_ and shear modulus *G*_R_, can be calculated from *C*_11_, *C*_22_, *C*_33_, *C*_12_, *C*_13_, *C*_23_, *C*_44_, *C*_55_, and C_66_ as [25]:


[4] $$B_{\text{V}}=\left(1/9\right)\left[C_{11}+C_{22}+C_{33}+2\left(C_{12}+C_{13}+C_{23}\right)\right],$$

[5] $$B_{\text{R}}=\Delta {\left[\begin{array}{l} C_{11}\left(C_{22}+C_{33}-2C_{23}\right)+C_{22}\left(C_{33}-2C_{13}\right)\\ -2C_{33}C_{12}+C_{12}\left(2C_{23}-C_{12}\right)\\ +C_{13}\left(2C_{12}-C_{13}\right)+C_{23}\left(2C_{13}-C_{23}\right) \end{array}\right]}^{-1},$$

[6] $$G_{\text{V}}=\left(1/15\right)\left[\begin{array}{l} C_{11}+C_{22}+C_{33}+3\left(C_{44}+C_{55}+C_{66}\right)\\ -\left(C_{12}+C_{13}+C_{23}\right) \end{array}\right],$$

[7] $$G_{\text{R}}\;=\;15{\left\{\begin{array}{l} 4\left[\begin{array}{l} C_{11}\left(C_{22}\;+\;C_{33}\;+\;C_{23}\right)+C_{22}\left(C_{33}\;+\;C_{13}\right)\\ +C_{33}C_{12}-C_{12}\left(C_{23}+C_{12}\right)\\ -C_{13}\left(C_{12}+C_{13}\right)-C_{23}\left(C_{13}+C_{23}\right) \end{array}\right]/\Delta \\ +3\left[\left(1/C_{44}\right)+\left(1/C_{55}\right)+\left(1/C_{66}\right)\right] \end{array}\right\}}^{-1}\text{​},$$

where


[8] $$\begin{array}{c} \Delta =C_{13}\left(C_{12}C_{23}-C_{13}C_{22}\right)+C_{23}\left(C_{12}C_{13}-C_{23}C_{11}\right)\\ +C_{33}\left(C_{11}C_{22}-C_{12}^{2}\right). \end{array}$$

The average bulk modulus *B* was calculated as the arithmetic average of *B*_V_ and *B*_R_, that is, *B* = (1/2)(*B*_V_ + *B*_R_). Likewise, the average shear modulus *G* was calculated by *G* = (1/2)(*G*_V_ + *G*_R_). From the bulk modulus *B* and the shear modulus *G*, the Young’s modulus *E* and the Poisson’s ratio ν can be calculated as [25]:


[9] E=9BG/(3B+G),

[10] ν=(3B−2G)/[2(3B+G)].

**Figure 2:**
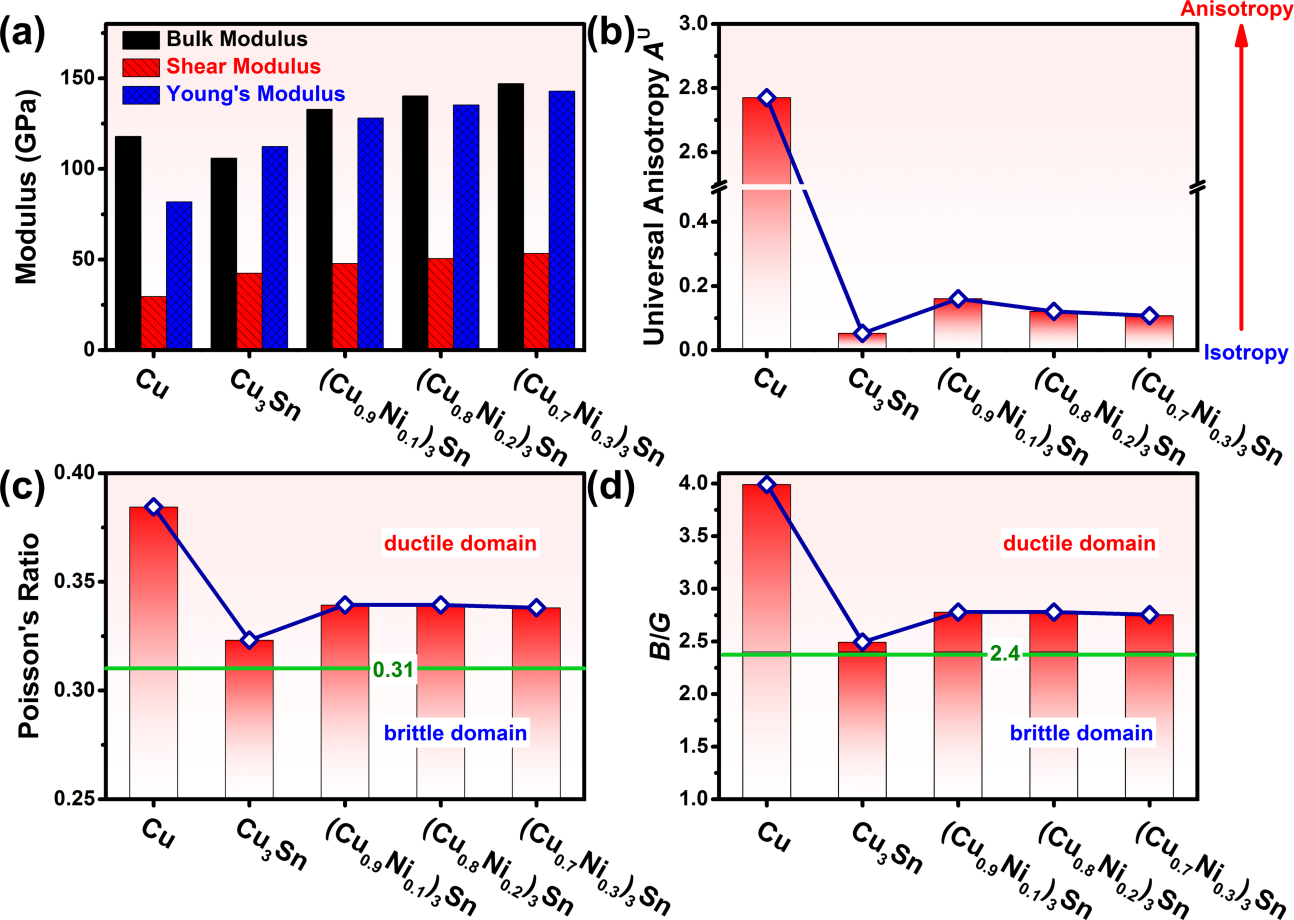
(a) Bulk moduli, shear moduli, and Young’s moduli of Cu and (Cu*_x_*Ni_1−_*_x_*)_3_Sn; (b) universal anisotropies *A*^U^ of Cu and (Cu*_x_*Ni_1−_*_x_*)_3_Sn; (c) Poisson’s ratios of Cu and (Cu*_x_*Ni_1−_*_x_*)_3_Sn; (d) ratios of bulk modulus to shear modulus, *B*/*G*, of Cu and (Cu*_x_*Ni_1−_*_x_*)_3_Sn. The green horizontal lines in (c) and (d) correspond to the boundaries of the ductile-to-brittle transition.

The calculated bulk moduli, shear moduli, and Young’s moduli of Cu and (Cu*_x_*Ni_1−_*_x_*)_3_Sn are presented in Figure 2a and listed in Table 2. As for Cu, the calculated bulk modulus of 117.9 GPa, the shear modulus of 29.5 GPa, and the Young’s modulus of 81.8 GPa are consistent with the experimental results [29]. Regarding the intermetallics (Cu*_x_*Ni_1−_*_x_*)_3_Sn, the bulk moduli, shear moduli, and Young’s moduli are greater than those of Cu, except the bulk modulus of Cu_3_Sn, which is less than that of Cu. At the same time, it can be observed that the bulk moduli, shear moduli, and Young’s moduli of (Cu*_x_*Ni_1−_*_x_*)_3_Sn increase with the increase of Ni content.

**Table 2:** Bulk modulus *B*, shear modulus *G*, Young’s modulus *E*, Poisson’s ratio ν; universal anisotropy *A*^U^; ductility index *B*/*G*, minimum Young’s modulus, *E*_min_ and the corresponding orientation [*l*_1_, *l*_2_, *l*_3_]_min_; as well as maximum Young’s modulus *E*_max_ and the corresponding orientation [*l*_1_, *l*_2_, *l*_3_]_max_. *l*_1_, *l*_2_, *l*_3_ are the direction cosines of the orientation axis. The data presented in parentheses are given for comparison.

	Cu	Cu_3_Sn	(Cu_0.9_Ni_0.1_)_3_Sn	(Cu_0.8_Ni_0.2_)_3_Sn	(Cu_0.7_Ni_0.3_)_3_Sn

*B* (GPa)	117.9 (140)^a^	105.9 (113.8)^b^	132.9	140.4	147.1
*G* (GPa)	29.5 (46.4)^a^	42.5 (46.7)^b^	47.9	50.5	53.4
*E* (GPa)	81.8 (112)^a^	112.3 (123.2)^b^	128.1	135.2	142.9
ν	0.384 (0.364)^a^	0.323 (0.319)^b^	0.339	0.339	0.338
*A* ^U^	2.770	0.052	0.160	0.121	0.107
*B*/*G*	4.00	2.49	2.78	2.78	2.76
*E*_min_ (GPa)	36.7	93.9	103.9	116.8	124.9
[*l*_1_, *l*_2_, *l*_3_]_min_	[1, 0, 0]	[1, 0, 0]	[1, 0, 0]	[1, 0, 0]	[1, 0, 0]
*E*_max_ (GPa)	134.8	118.6	147.9	161.8	177.8
[*l*_1_, *l*_2_, *l*_3_]_max_	[0.5774, 0.5774, 0.5774]	[0.5918, 0.5137, 0.6212]	[0.2978, 0.0000, 0.9546]	[0, 0, 1]	[0, 0, 1]

^a^The experimental elastic moduli and Poisson’s ratios of Cu are cited from [29]. ^b^The experimental elastic moduli and Poisson’s ratios of Cu_3_Sn are cited from [27].

Moreover, using the calculated values of *B*_V_, *B*_R_, *G*_V_, and *G*_R_, the universal anisotropy index *A*^U^, developed by Ostoja-Starzewski et al., can be expressed as [30]:


[11] $$A^{\text{U}}=5\frac{G_{\text{V}}}{G_{\text{R}}}+\frac{B_{\text{V}}}{B_{\text{R}}}-6.$$

Because the mechanical response of crystals to external loads strongly depends on the elastic anisotropy, the anisotropies of Cu and (Cu*_x_*Ni_1−_*_x_*)_3_Sn were evaluated by the universal anisotropy index, *A*^U^, which was calculated according Equation 11. In general, for locally isotropic crystals *A*^U^ = 0. As the elastic anisotropy of the crystal increases, the value of *A*^U^ will increase correspondingly. As shown in Figure 2b, the *A*^U^ of Cu is 2.77. It indicates that Cu is intrinsically anisotropic, although Cu has a FCC structure. In contrast to the anisotropy of Cu, the *A*^U^ of orthorhombic Cu_3_Sn is 0.052. This implies that Cu_3_Sn is locally elastically isotropic. After Ni alloying, the *A*^U^ of (Cu_0.9_Ni_0.1_)_3_Sn increases to 0.160. With a further increase of Ni content, the *A*^U^ of (Cu*_x_*Ni_1−_*_x_*)_3_Sn decreases from 0.121 in (Cu_0.8_Ni_0.2_)_3_Sn to 0.107 in (Cu_0.7_Ni_0.3_)_3_Sn. This demonstrates that the orthorhombic (Cu*_x_*Ni_1−_*_x_*)_3_Sn compounds are more isotropic than FCC Cu.

Besides elastic modulus and the anisotropy, the intrinsic ductilities of Cu and (Cu*_x_*Ni_1−_*_x_*)_3_Sn were evaluated using the parameters ν and *B*/*G*. Poisson’s ratio ν is defined as the ratio of the transverse strain to the longitudinal strain, and it characterizes the resistance of materials to distortion under mechanical load [31]. The boundary of the ductile-to-brittle transition (DTB) can be identified as ν = 0.31 [31]. As presented in Figure 2c, the calculated Poisson’s ratio of Cu is 0.384, which is in good agreement with the experimental value of 0.364 [29]. It reveals that Cu is an intrinsically ductile metal. For Cu_3_Sn, the calculated Poisson’s ratio is 0.323, which is in the vicinity of the DTB boundary. This indicates that the intrinsic ductility of Cu_3_Sn is poor. However, by Ni alloying, the Poisson’s ratio of (Cu_0.9_Ni_0.1_)_3_Sn increases up to 0.339. With further increase of Ni content, the Poisson’s ratio of (Cu*_x_*Ni_1−_*_x_*)_3_Sn levels off about 0.339. In comparison with Cu_3_Sn, the increasing Poisson’s ratio indicates that improved ductility of (Cu*_x_*Ni_1−_*_x_*)_3_Sn can be achieved by Ni alloying.

In addition to Poisson’s ratio, another ductility index, that is, the ratio of bulk modulus to shear modulus, *B*/*G*, was adopted to evaluate the ductilities of Cu and (Cu*_x_*Ni_1−_*_x_*)_3_Sn. *B*/*G* is a measure of the plastic flow at the crack tip; low *B*/*G* values will lead to difficulties of plastic flow and, correspondingly, result in brittle behavior. In contrast, high *B*/*G* values give rise to an ease of the plastic flow and result in ductile behavior [32]. In terms of *B*/*G*, the boundary of DTB can be identified as *B*/*G* = 2.4 [31]. As presented in Figure 2d, the *B*/*G* value of Cu is 4.0, which is obviously higher than the DTB boundary of 2.4. This indicates the intrinsic ductility of Cu. The *B*/*G* value of Cu_3_Sn is 2.49, which is close to the DTB boundary of 2.4. It manifests the poor ductility of Cu_3_Sn. After Ni alloying of Cu_3_Sn, the *B*/*G* value of (Cu_0.9_Ni_0.1_)_3_Sn increases to 2.78. With the further increase of Ni content, the *B*/*G* of (Cu*_x_*Ni_1−_*_x_*)_3_Sn levels off about 2.78. The increasing values of *B*/*G* indicate that the ductility of (Cu*_x_*Ni_1−_*_x_*)_3_Sn is improved by Ni alloying. The ductility evaluations of Cu and (Cu*_x_*Ni_1−_*_x_*)_3_Sn in terms of Poisson’s ratio and *B*/*G* are mutually consistent.

### Orientation-dependent Young’s moduli of Cu and (Cu*_x_*Ni_1−_*_x_*)_3_Sn

Considering the critical roles of orientation-dependent elastic properties in the mechanical properties of interfaces [19], the orientation-dependent Young’s moduli of Cu and (Cu*_x_*Ni_1−_*_x_*)_3_Sn were investigated. Regarding FCC Cu, the orientation-dependent Young’s moduli,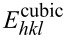, along the directions ⟨*hkl*⟩ were calculated as [33]:


[12] $$\frac{1}{E_{hkl}^{\text{cubic}}}=s_{11}-2\left[\left(s_{11}-s_{12}\right)-\frac{1}{2}s_{44}\right]\left(l_{1}^{2}l_{2}^{2}+l_{2}^{2}l_{3}^{2}+l_{1}^{2}l_{3}^{2}\right).$$

**Figure 3:**
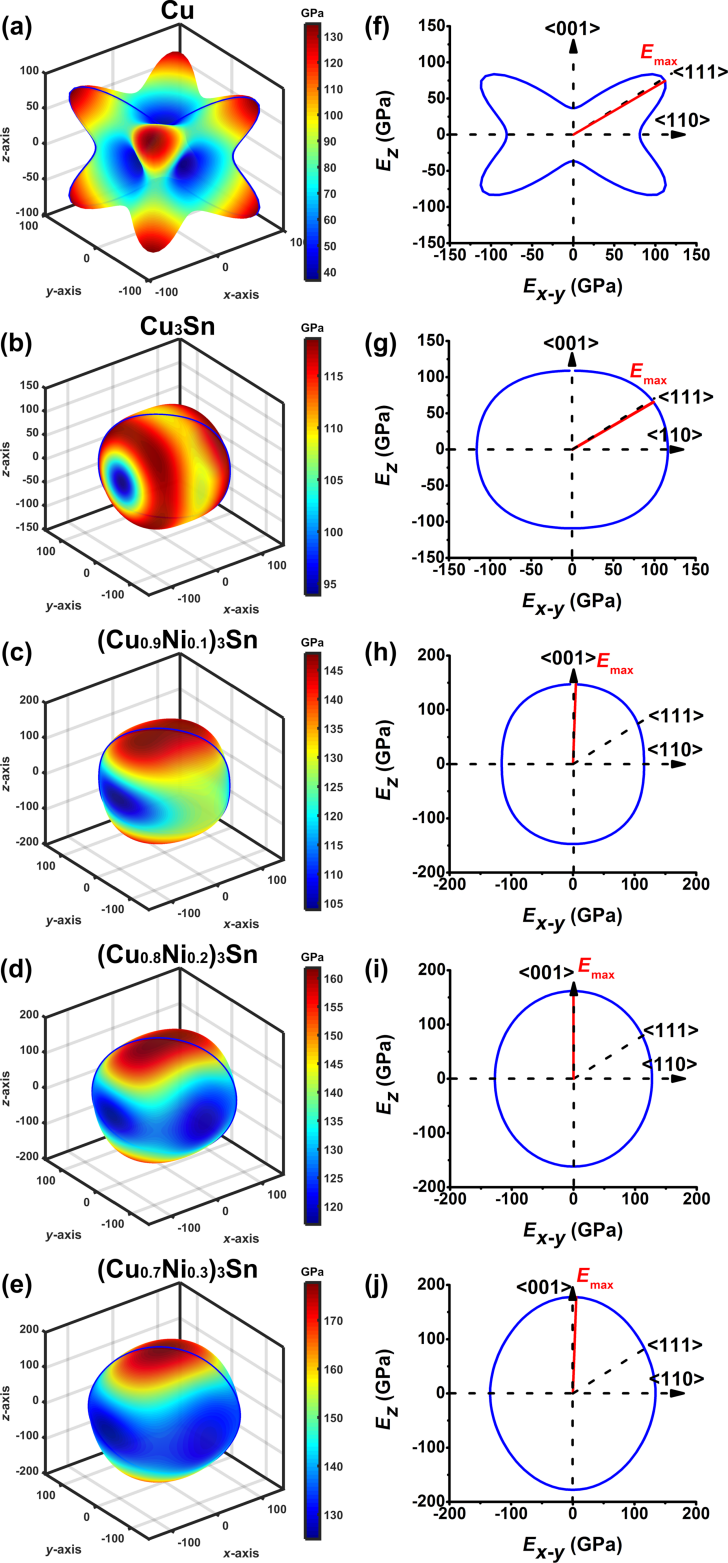
3D surfaces of oriented Young’s moduli for (a) Cu, (b) Cu_3_Sn, (c) (Cu_0.9_Ni_0.1_)_3_Sn, (d) (Cu_0.8_Ni_0.2_)_3_Sn, and (e) (Cu_0.7_Ni_0.3_)_3_Sn. 2D profiles on the (110) plane of oriented Young’s moduli for (f) Cu, (g) Cu_3_Sn, (h) (Cu_0.9_Ni_0.1_)_3_Sn, (i) (Cu_0.8_Ni_0.2_)_3_Sn, and (j) (Cu_0.7_Ni_0.3_)_3_Sn; the orientations of the maximum Young’s moduli in the 2D profiles are denoted by the red lines.

For orthorhombic (Cu*_x_*Ni_1−_*_x_*)_3_Sn, the orientation-dependent Young’s moduli, 
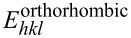
, along the directions ⟨*hkl*⟩ were calculated as [33]:


[13] $$\begin{array}{c} \frac{1}{E_{hkl}^{\text{orthorhombic}}}=l_{1}^{4}s_{11}+l_{2}^{4}s_{22}+l_{3}^{4}s_{33}+2l_{1}^{2}l_{2}^{2}s_{12}+2l_{1}^{2}l_{3}^{2}s_{13}\\ +2l_{2}^{2}l_{3}^{2}s_{23}+l_{2}^{2}l_{3}^{2}s_{44}+l_{1}^{2}l_{3}^{2}s_{55}+l_{1}^{2}l_{2}^{2}s_{66}, \end{array}$$

where *s*_11_, *s*_22_, *s*_33_, *s*_12_, *s*_13_, *s*_23_, *s*_44_, *s*_55_, and *s*_66_ are the elements of the elastic compliance matrix. The elastic compliance matrix is the inverse of the elastic constant matrix. *l*_1_, *l*_2_ and *l*_3_ are the direction cosines of the ⟨*hkl*⟩ axes. Using the elastic compliance matrix, the three-dimensional (3D) oriented Young’s moduli of Cu and (Cu*_x_*Ni_1−_*_x_*)_3_Sn were calculated using Equation 12 and Equation 13, respectively. Maximum and minimum of Young’s moduli and the corresponding orientations are listed in Table 2. As shown in Figure 3a, the orientation-dependent Young’s moduli of FCC Cu exhibit a star-shaped surface, and the maximum Young’s modulus is oriented along the body diagonal, that is, the ⟨111⟩ direction. In contrast, as shown in Figure 3b–e, the orientation-dependent Young’s moduli of (Cu*_x_*Ni_1−_*_x_*)_3_Sn exhibit a spheroidal surface. After Ni alloying, the spheroidal surfaces of (Cu*_x_*Ni_1−_*_x_*)_3_Sn are expanded. As the Ni content increases, the direction of the maximum Young’s modulus changes from off the *z*-axis in Cu_3_Sn to along the *z*-axis in (Cu_0.9_Ni_0.1_)_3_Sn, (Cu_0.8_Ni_0.2_)_3_Sn, and (Cu_0.7_Ni_0.3_)_3_Sn. The shapes of the 3D oriented Young’s moduli demonstrate that the anisotropy of FCC Cu is higher than those of orthorhombic (Cu*_x_*Ni_1−_*_x_*)_3_Sn. The anisotropies of Cu and (Cu*_x_*Ni_1−_*_x_*)_3_Sn revealed by the 3D orientation-dependent Young’s moduli are consistent with the anisotropies measured by the universal anisotropy *A*^U^.

By cutting the 3D oriented Young’s moduli in the (110) plane, along the solid lines shown in Figure 3a–e, specific magnitude and direction of the maximum Young’s moduli of Cu and (Cu*_x_*Ni_1−_*_x_*)_3_Sn were analyzed. The magnitude of the maximum Young’s modulus *E*_max_ is given by 
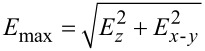
. The 2D Young’s modulus for Cu is shown in Figure 3f. It can be seen that the orientation-dependent Young’s modulus of Cu shows a fourfold petal-shaped profile, the maximum Young’s modulus of 134.8 GPa is along the ⟨111⟩ direction. As shown in Figure 3g, the maximum Young’s modulus of Cu_3_Sn is 118.6 GPa with orientation close to the ⟨111⟩ direction. Figure 3h–j shows that the maximum Young’s moduli of (Cu_0.9_Ni_0.1_)_3_Sn, (Cu_0.8_Ni_0.2_)_3_Sn, and (Cu_0.7_Ni_0.3_)_3_Sn are 147.9, 161.8, and 177.8 GPa, respectively, with orientation close to the ⟨001⟩ direction. These results reveal that the Ni alloying in (Cu*_x_*Ni_1−_*_x_*)_3_Sn will not only increase the magnitude of the maximum Young’s modulus, but also turns the orientation of the maximum Young’s modulus from off the *z*-axis to along the *z*-axis.

### Interfacial mechanical properties of (Cu*_x_*Ni_1−_*_x_*)_3_Sn/Cu

**Figure 4:**
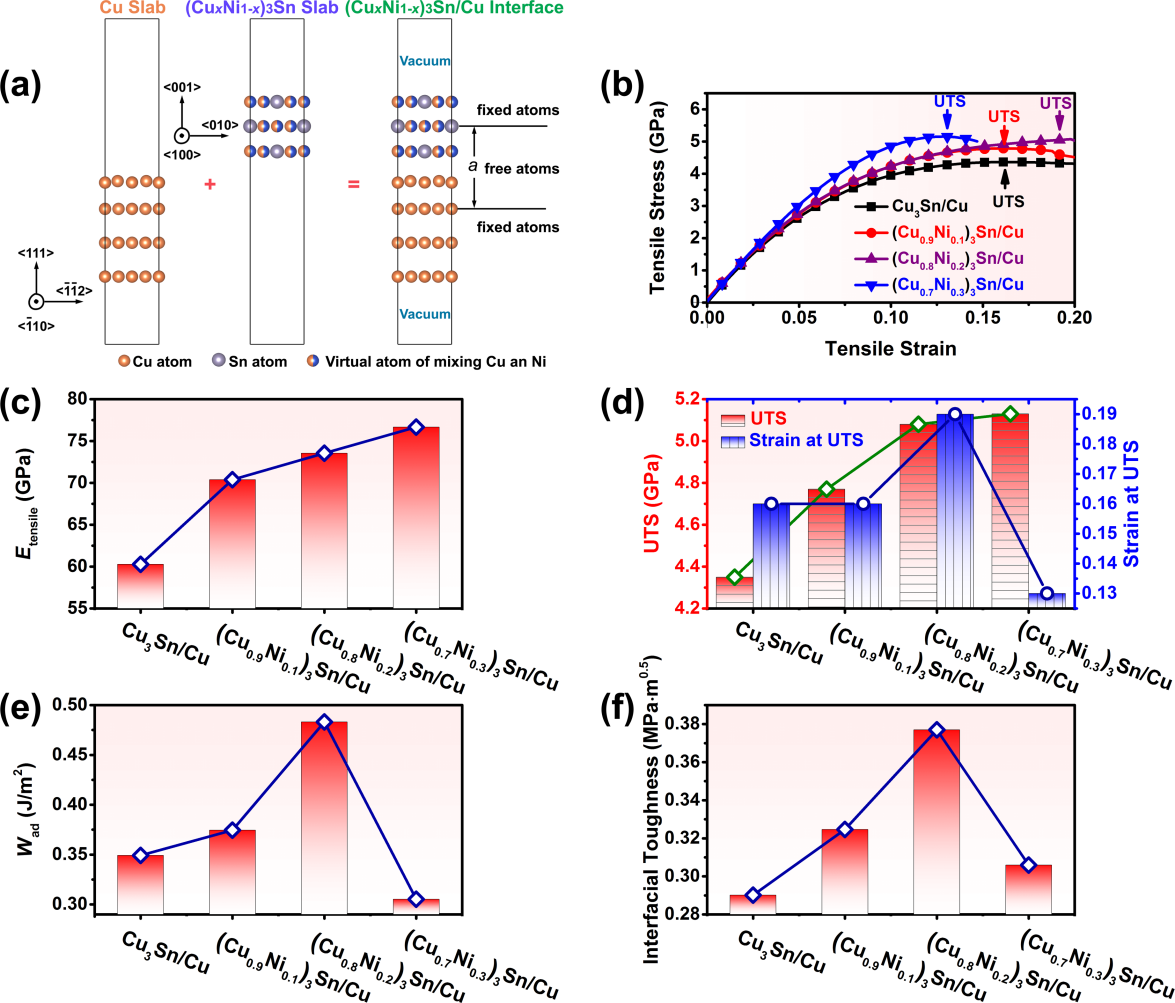
(a) Orientation relationship and structure of (Cu*_x_*Ni_1−_*_x_*)_3_Sn/Cu interfaces; the atomic models were plotted using VESTA [28]. This content is not subject to CC BY 4.0; (b) tensile stress vs tensile strain curves of (Cu*_x_*Ni_1−_*_x_*)_3_Sn/Cu interfaces; (c) tensile moduli *E*_tensile_ of (Cu*_x_*Ni_1−_*_x_*)_3_Sn/Cu interfaces; (d) ultimate tensile strengths UTS and tensile strains at UTS of (Cu*_x_*Ni_1−_*_x_*)_3_Sn/Cu interfaces; (e) work of adhesion *W*_ad_ of (Cu*_x_*Ni_1−_*_x_*)_3_Sn/Cu interfaces; (f) interfacial toughness of (Cu*_x_*Ni_1−_*_x_*)_3_Sn/Cu interfaces.

In the following, the mechanical properties of (Cu*_x_*Ni_1−_*_x_*)_3_Sn/Cu interfaces were investigated. As shown in Figure 4a, the interface structure of (Cu*_x_*Ni_1−_*_x_*)_3_Sn/Cu was modelled as a (Cu*_x_*Ni_1−_*_x_*)_3_Sn slab with three atomic layers, a Cu slab with four atomic layers, and a vacuum layer with thickness of 1 nm. The adjacent atomic layers of Cu slab and (Cu*_x_*Ni_1−_*_x_*)_3_Sn slab were free, and the remaining atomic layers of Cu slab and (Cu*_x_*Ni_1−_*_x_*)_3_Sn slab were fixed. The thickness of the interface layer was defined as thickness of free atomic layers, *a*. Tensile stress was applied along the direction normal to the interface plane, and the relationship between tensile stress and tensile strain of (Cu*_x_*Ni_1−_*_x_*)_3_Sn/Cu interfaces were calculated (Figure 4b). The tensile modulus, *E*_tensile_, was determined as the ratio of the tensile stress to the corresponding strain in the tensile strain range from 0.00 to 0.03. As shown in Figure 4c, the tensile moduli of (Cu*_x_*Ni_1−_*_x_*)_3_Sn/Cu interfaces increase from 60.3 GPa in Cu_3_Sn/Cu to 76.7 GPa in (Cu_0.7_Ni_0.3_)_3_Sn/Cu. The increase of the tensile modulus can be attributed to the fact that the Young’s moduli of (Cu*_x_*Ni_1−_*_x_*)_3_Sn along the ⟨001⟩ direction increase with Ni content.

From the tensile stress vs tensile strain curves, the ultimate tensile strength, UTS, and the corresponding tensile strain were determined, where UTS corresponds to the maximum tensile stress that the interface structures can endure. As shown in Figure 4d, the UTS of (Cu*_x_*Ni_1−_*_x_*)_3_Sn/Cu interfaces increase from 4.35 GPa in Cu_3_Sn/Cu to 5.13 GPa in (Cu_0.7_Ni_0.3_)_3_Sn/Cu. The tensile strain corresponding to the UTS of (Cu*_x_*Ni_1−_*_x_*)_3_Sn/Cu interfaces increases from the 0.16 in Cu_3_Sn/Cu to 0.19 in (Cu_0.8_Ni_0.2_)_3_Sn/Cu. However, with the further increase of Ni content, the tensile strain corresponding to the UTS of (Cu_0.7_Ni_0.3_)_3_Sn/Cu decreases to 0.13.

As far as the interfacial stability is concerned, it is generally characterized by the work of adhesion, that is, the work required to separate the interface structure into two parts [34]. The UTS corresponds to the maximum stress that the interface structure can endure, in other words, the UTS corresponds to the onset of interface structure destabilization. Thus, the bulk energy density stored in the interface structure can be calculated by integrating the product of tensile stress and tensile strain from tensile strain at zero stress to the strain at UTS. Then, the work of adhesion can be calculated by the product of bulk energy density and thickness of the interface layer, namely, the areal energy density stored during the tensile deformation before the structure reached destabilization [19]:


[14] $$W_{\text{ad}}\text{=}a\cdot \int_{0}^{\text{strain at UTS}}\sigma \cdot \varepsilon \text{ d}\varepsilon ,$$

where *a* is the thickness of the interface layer, σ is the tensile stress, and ε is the tensile strain. As shown in Figure 4e, the calculated *W*_ad_ values of Cu_3_Sn/Cu, (Cu_0.9_Ni_0.1_)_3_Sn/Cu, (Cu_0.8_Ni_0.2_)_3_Sn/Cu, and (Cu_0.7_Ni_0.3_)_3_Sn/Cu are 0.349, 0.374, 0.483, 0.305 J·m^−2^, respectively. The maximum value of *W*_ad_ = 0.483 J·m^−2^ was obtained in (Cu_0.8_Ni_0.2_)_3_Sn/Cu; it can be attributed to the high UTS and the large tensile strain at UTS.

From the calculated work of adhesion *W*_ad_ and tensile modulus *E*_tensile_, the interfacial toughness of (Cu*_x_*Ni_1−_*_x_*)_3_Sn/Cu can be calculated as follows [34]:


[15] $$K_{\text{IC}}^{\text{interface}}=\sqrt{4W_{\text{ad}}E_{\text{tensile}}}/10^{6},$$

where 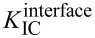 is the interfacial toughness. As presented in Figure 4f and listed in Table 3, the calculated interfacial toughnesses of Cu_3_Sn/Cu, (Cu_0.9_Ni_0.1_)_3_Sn/Cu, (Cu_0.8_Ni_0.2_)_3_Sn/Cu, and (Cu_0.7_Ni_0.3_)_3_Sn/Cu are 0.290, 0.325, 0.377, and 0.306 MPa·m^0.5^, respectively. It can be seen that the interfacial toughness of (Cu*_x_*Ni_1−_*_x_*)_3_Sn/Cu increases with increasing Ni content, as the composition changes from Cu_3_Sn/Cu to (Cu_0.8_Ni_0.2_)_3_Sn/Cu. After further increase of Ni content, the interfacial toughness will decrease to 0.306 MPa·m^0.5^ for (Cu_0.7_Ni_0.3_)_3_Sn/Cu.

**Table 3:** Thickness of interface layer *a*, tensile modulus *E*_tensile_, ultimate tensile stress UTS, strain at UTS, work of adhesion *W*_ad_, and interfacial toughness 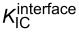 of (Cu*_x_*Ni_1−_*_x_*)_3_Sn/Cu interfaces.

	Cu_3_Sn/Cu	(Cu_0.9_Ni_0.1_)_3_Sn/Cu	(Cu_0.8_Ni_0.2_)_3_Sn/Cu	(Cu_0.7_Ni_0.3_)_3_Sn/Cu

*a* [m]	6.96 × 10^−10^	6.96 × 10^−10^	6.96 × 10^−10^	6.96 × 10^−10^
*E*_tensile_ [Pa]	6.03 × 10^10^	7.04 × 10^10^	7.35 × 10^10^	7.67 × 10^10^
UTS [GPa]	4.35	4.77	5.08	5.13
strain at UTS	0.16	0.16	0.19	0.13
*W*_ad_ [J·m^−2^]	0.349	0.374	0.483	0.305
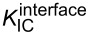 [MPa·m^0.5^]	0.290	0.325	0.377	0.306

In the following, the mechanisms responsible for the effects of Ni alloying on the interfacial mechanical properties of (Cu*_x_*Ni_1−_*_x_*)_3_Sn/Cu are discussed. The interfacial toughness increases with the Ni content when the composition changes from Cu_3_Sn/Cu to (Cu_0.8_Ni_0.2_)_3_Sn/Cu. Such improvement can be related to the enhanced oriented Young’s modulus and ductility of (Cu*_x_*Ni_1−_*_x_*)_3_Sn. The oriented Young’s modulus of (Cu*_x_*Ni_1−_*_x_*)_3_Sn along the ⟨001⟩ direction increases from 109.0 GPa in Cu_3_Sn to 161.8 GPa in (Cu_0.8_Ni_0.2_)_3_Sn. At the same time, the ductility of (Cu*_x_*Ni_1−_*_x_*)_3_Sn, in terms of *B*/*G* index, increases from 2.49 in Cu_3_Sn to 2.78 in (Cu_0.8_Ni_0.2_)_3_Sn. Owing to enhanced oriented Young’s modulus and ductility, both UTS and tensile strain at UTS increase as the composition changes from Cu_3_Sn/Cu to (Cu_0.8_Ni_0.2_)_3_Sn/Cu. The enhanced UTS and tensile strain at UTS will lead to an improvement of the work of adhesion and, in turn, result in a reinforced interfacial toughness.

The deterioration of the interfacial toughness of (Cu_0.7_Ni_0.3_)_3_Sn/Cu with high Ni content can be attributed to the higher oriented Young’s modulus and smaller strain at UTS. Considering the fact that the ductility of (Cu*_x_*Ni_1−_*_x_*)_3_Sn levels off as the Ni content increases, the higher UTS of (Cu_0.7_Ni_0.3_)_3_Sn will result in the premature destabilization of the interface layer and reduced tensile strain at UTS. Although the UTS increases, the lower tensile strain at UTS severely reduces the work of adhesion and, in turn, decreases the interfacial toughness. This implies that the enhanced interfacial toughness originates from the simultaneous increase of the oriented Young’s modulus and the ductility. Increasing only the Young’s modulus may even worsen the interfacial toughness. Moreover, regarding that (Cu*_x_*Ni_1−_*_x_*)_3_Sn is thermodynamically stable in the range *x* = 0.72–1.00 [22], another factor that causes the deterioration of the interfacial toughness of (Cu_0.7_Ni_0.3_)_3_Sn/Cu may be the thermodynamic stability of (Cu_0.7_Ni_0.3_)_3_Sn. Basically, within the thermodynamically stable domain of (Cu*_x_*Ni_1−_*_x_*)_3_Sn, the interfacial toughness of (Cu*_x_*Ni_1−_*_x_*)_3_Sn/Cu increases with the increase of Ni content is dominated by mechanical mechanisms. In contrast, for the case of high Ni content, the deterioration of the interfacial toughness may be caused by mechanical or thermodynamic mechanisms.

## Conclusion

First, the calculated elastic properties of Cu and (Cu*_x_*Ni_1−_*_x_*)_3_Sn reveal that the Ni alloying in (Cu*_x_*Ni_1−_*_x_*)_3_Sn can effectively improve the pure tensile elastic constants and orthogonal elastic constants; however, the alloying only slightly improves the pure shear elastic constants. The improvement of elastic constants by Ni alloying can result in the improvement of elastic modulus and ductility of (Cu*_x_*Ni_1−_*_x_*)_3_Sn with the increase of Ni content.

Second, the calculated oriented Young’s moduli of Cu and (Cu*_x_*Ni_1−_*_x_*)_3_Sn reveal that the anisotropy of FCC Cu is higher than those of the orthorhombic (Cu*_x_*Ni_1−_*_x_*)_3_Sn intermetallics. The maximum Young’s modulus of Cu is oriented along the ⟨111⟩ direction. However, with the increase of Ni content, the orientation of the maximum Young’s moduli of (Cu*_x_*Ni_1−_*_x_*)_3_Sn change from the ⟨111⟩ direction of Cu_3_Sn to the ⟨001⟩ direction of (Cu_0.7_Ni_0.3_)_3_Sn.

Finally, according to the orientation relationship of the (Cu*_x_*Ni_1−_*_x_*)_3_Sn/Cu interface, the calculated tensile stress vs strain curves of (Cu*_x_*Ni_1−_*_x_*)_3_Sn/Cu interfaces reveal that the tensile moduli and UTS monotonically increase with the increase of Ni content. The work of adhesion and interfacial toughness increase with the increase of Ni content within the thermodynamically stable domain of (Cu*_x_*Ni_1−_*_x_*)_3_Sn; thus, the mechanism responsible for the enhancement of interfacial mechanical properties can be attributed to the simultaneous improvements of oriented Young’s modulus and ductility of (Cu*_x_*Ni_1−_*_x_*)_3_Sn achieved by Ni alloying. With the further increase of Ni content beyond the thermodynamically stable domain, the work of adhesion and interfacial toughness of (Cu*_x_*Ni_1−_*_x_*)_3_Sn/Cu interfaces will deteriorate. This deterioration through high Ni content can be attributed to the premature destabilization of the interface structure owing to the higher oriented Young’s modulus and smaller strain at UTS of (Cu*_x_*Ni_1−_*_x_*)_3_Sn. In addition, the thermodynamically instability of (Cu*_x_*Ni_1−_*_x_*)_3_Sn may also deteriorate the work of adhesion and interfacial toughness.

Overall, within the thermodynamically stable domain of (Cu*_x_*Ni_1−_*_x_*)_3_Sn, the increase of Ni content can enhance the interfacial mechanical properties of (Cu*_x_*Ni_1−_*_x_*)_3_Sn/Cu and increase the reliability of the lead-free solder joints. But higher Ni content beyond the thermodynamically stable domain of (Cu*_x_*Ni_1−_*_x_*)_3_Sn will deteriorate the interfacial mechanical properties by mechanical or thermodynamic mechanisms and decrease the reliability of the lead-free solder joints.

## Data Availability

All data that supports the findings of this study is available in the published article.
